# Simulation of tight fluid flow with the consideration of capillarity and stress-change effect

**DOI:** 10.1038/s41598-019-41861-3

**Published:** 2019-03-29

**Authors:** Yuan Zhang, Yuan Di, Pengcheng Liu, Wanzhen Li

**Affiliations:** 1 School of Energy Resources, China University of Geosciences (Beijing), Xueyuan Rd. 29#, Haidian district, Beijing, 100083 China; 2 Beijing Key Laboratory of Unconventional Natural Gas Geology Evaluation and Development Engineering, Xueyuan Rd. 29#, Haidian district, Beijing, 100083 China; 3 College of Engineering, Peking University, Yiheyuan Rd. 5#, Haidian district, Beijing, 100871 China

## Abstract

The horizontal wells and multi-stage hydraulic fracturing technologies play a significantly important role in developing unconventional reservoirs. Due to the nanopore effects and stress deformation in tight formations, the fluid equilibrium and thermodynamics become more complex and the conventional reservoir simulation models cannot accurately handle these mechanisms. Hence, the objective of this work is to propose a comprehensive simulation model considering the effects of confined space and stress-dependent deformation. We first evaluated the phase envelope and fluid properties in the confined nanopores. Results show that bubble-point pressure and oil viscosity decrease, while formation volume factor and gas-oil ratio increase. The heavy components cause large deviation on the P-T phase envelope at the reservoir condition. Subsequently, a reservoir simulation model of the Bakken tight oil reservoir was built including the effect of stress-dependent deformation. The proposed phase behavior model was applied into the reservoir simulator to predict the hydrocarbon production from the Bakken tight oil reservoir. Finally, the role of the confined space and the stress-dependent deformation on the production are examined in detail. This novel simulation approach can shed light on the better understanding of the key parameters affecting well production of in developing tight oil reservoirs in the future.

## Introduction

Unconventional reservoirs, including tight oil, shale gas, and tight gas are key to meeting the increasing demand for hydrocarbon fuels in the world^1,2^. Tight oil reservoirs have recently become a hotspot for development in unconventional oil and gas resources^3,4^. As reported, the production of tight oil reservoirs will increase to more than double from the 2015 to the 2040 all over the world. Hydraulic fracturing has played a prominent role in the improvement of the oil and gas production^5^. Due to the characteristics of low permeability and low porosity, the combination of horizontal wells and multi-stage hydraulic fracturing has been applied to improve the contacted volume and flow capacity in developing tight oil reservoirs^6–9^. In spite of great achievement, the mechanisms such as confinement in nanopores and geomechanics are still not well understood^10,11^.

Recent studies have shown that phase behavior and fluid transport exhibit deviation from the bulk fluid due to the nanopores in tight oil reservoirs. Sigmund *et al*. demonstrated that the effects of curvature cannot be neglected at high surface curvatures^12^. Brusilovsky investigated the phase behavior of binary mixture experimentally, concluding that the dew point pressure increases because of the surface curvature^13^. Nojabaei *et al*. also pointed out that the capillary pressure leads to the significant suppression on the bubble point pressure^14^.

Similar conclusions were also reported in others literatures^15–21^. In Nojabaei *et al*.’s work, they then developed a compositionally-extended black-oil simulator to evaluate the capillarity effect on the well production^14^. However, pore characterization and geomechanics were not considered. Teklu *et al*. observed the minimum miscibility pressure (MMP) decreases in the confined space^22^. Luo *et al*. conducted experiments of phase behavior in nanopores and also pointed out that the bubble point pressure of confined fluid deviates from the bulk fluid^23^. Equation of state, such as Peng-Robinson (PR) and Perturbed-Chain Statistical Associating Fluid Theory (PC-SAFT) was implemented to represent the fluid-phase equilibria in nanosized pores^24–26^. Dong *et al*. systemically analyzed the capillary pressure effect, adsorption and the critical shifts on the behavior of the confined fluid^27^. They found that bubble-point pressure is overestimated if neglecting the adsorption film. Haider and Aziz explored the key parameters of nano-porous media in shale reservoirs^28^. Their findings showed that the fluid properties and well production are both influenced in the confined space. Cui *et al*. modified the Peng-Robinson equation of state considering the reduced mole number of fluids caused by adsorption^29^. Zuo *et al*. first applied SRK-EOS for the phase behavior calculations in the confined phase and investigated the impact of capillary pressure and nanopore confinement on the phase behaviors of shale gas and oil^30^. These methods are mainly based on the pressure-temperature flash calculation, which uses the pressure, temperature, and fluid composition as the primal state variables (NVT-flash)^31–35^. An algorithm for calculating phase equilibrium at specified moles, volume, and temperature was proposed in recent years; additionally, Kou and Sun incorporated the capillarity into the NVT-flash. This method can be used to predict the phase properties of pure substance and mixture systems^36^.

The impacts of the physical mechanisms on the well performance have received more attention. Du and Chu^17^ used a commercial reservoir simulator to investigate the influences of PVT variations on well performance. However, they only considered the single pore size and assumed the matrix permeability as a fixed value. Considering the change of porosity and permeability with effective stress, Wang *et al*. concluded that the well performance was affected due to the capillary pressure in the nanopores^37^. Nojabaei *et al*. took the pore size distribution into account and incorporated the capillarity effect in their simulation model, but the variation of permeability and stress sensitivity were not included^38^. Sanaei *et al*. developed a correlation between pore size and permeability based on mercury injection capillary pressure (MICP) tests on Eagle Ford core plugs, but there are still some limits on the field-scale application^39^. Rezaveisi *et al*. implemented the capillarity equilibrium in an in-house simulator and observed obvious difference on the production^40^. However, stress sensitivity was not considered in their model. Yan *et al*. developed a fully compositional model considering the effect of nanopores, in their approach, capillary pressure is calculated by Leverett J-function^41^. Siripatrachai *et al*. built an in-house compositional simulator to investigate the capillary pressure effect on the production of hydraulically fractured horizontal well^42^. Yu and Sepehrnoori (2018) concluded the capillary pressure effect on the well performance with the complex fracture geometries^43^. However, the physical mechanisms are not included in their study.

Our objective is to build a more efficient method to comprehensively evaluate the well performance considering the effect of capillary pressure on phase behavior and stress-change effect, especially for the field-scale case study by incorporating the flash calculation and the commercial simulators. We combine the phase behavior calculation with the CMG commercial simulator, which offers more chances to investigate important factors affecting well production of tight oil reservoirs. In this work, the phase equilibrium calculation considering the confined space was included, which has been validated against the experimental data^44^. The impact of capillary pressure on phase behavior was investigated for different compositions. Subsequently, a field-scale simulation model was built including two horizontal wells with hydraulic fractures from the Bakken tight oil reservoir. We performed analysis of different LGR-grid cells to reduce the computational cost and numerical dispersion. Furthermore, the black-oil properties were calculated using the proposed phase behavior model, which were then implemented in the black-oil reservoir simulator. Finally, the field-scale case was conducted to evaluate the effects of confined space and tress-dependent deformation on the well production. Results illustrate that the phase equilibrium in the confined space facilitates the oil production, while the stress-dependent deformation offsets the well performance. This novel simulation approach can provide a useful tool to predict the well performances in developing tight oil reservoirs.

## Methodology

### Fluid flow mechanism

The governing equation was applied to model the total mass balance for a component “*i*” in the oil and gas phases^45^.

1 $$\frac{\partial }{\partial t}(\phi \sum _{l=1}^{{N}_{p}}{\rho }_{l}{S}_{l}{w}_{il})+\overrightarrow{\nabla }\cdot (\sum _{l=1}^{{N}_{p}}{\rho }_{l}{w}_{il}{u}_{l}-\phi {\rho }_{l}{S}_{l}{\overline{\overline{K}}}_{il}\nabla {w}_{il})-{r}_{i}=0,\,i=1,\ldots ,{N}_{p}$$

where *φ* denotes porosity, *ρ*_*l*_ and *S*_*l*_ are density and saturation of phase *l*, *r*_*i*_ is mass rate injection or production as source or sink term. *N*_*p*_ is number of phase, *w*_*il*_ is mass fraction of component *i* in the phase *l* per unit volume. *u*_*l*_ is Darcy’s flow velocity and ${\overline{\overline{K}}}_{il}$ is the dispersivity coefficient of component *i* in the phase *l*.

*u*_*l*_ and ${\overline{\overline{K}}}_{il}$ can be described as Eqs (2) and (3):

2 $${\overline{u}}_{l}=-\,\frac{\overline{\overline{k}}}{{\mu }_{l}}(\overline{\nabla }{P}_{l}-{\rho }_{l}\overline{g}),$$

where $\overline{\overline{k}}$ is permeability tensor, *P*_*l*_ is pressure of phase *l* and *μ*_*l*_ is viscosity of phase *l*.

3 $${\overline{\overline{K}}}_{il}=\frac{{\overline{\overline{D}}}_{il}}{\tau }+\frac{{\overline{\alpha }}_{l}|{\overline{u}}_{l}|}{\phi {S}_{l}}$$

where ${\overline{\alpha }}_{l}$ is the dispersivity coefficient of phase *l* in the longitudinal direction and two transverse directions, *τ* is tortuosity, ${\overline{\overline{D}}}_{il}$ is the diffusion coefficient of component *i* in the phase *l*, which can be taken from work by Sigmund^46^.

In this study, these equations are solved using CMG-IMEX module, a numerical black-oil reservoir simulator^47^.

### Vapor-liquid phase equilibrium in the confined space

The fugacities of the liquid and vapor phases at equilibrium can be expressed as^48^,

4 $${f}_{L}^{i}(T,{P}_{L},{x}_{i})={f}_{V}^{i}(T,{P}_{V},{y}_{i}),\,i=1,\ldots ,{N}_{c},$$

where *f*^*i*^ is the fugacity of component *i* in the liquid and vapor phases. *P*_*V*_ and *P*_*L*_ refer to the pressures in the vapor and liquid phase, respectively. Due to the interactions in the confined space, *P*_*V*_ is not equal to *P*_*L*_, and it is defined as^48^:

5 $${P}_{V}-{P}_{L}={P}_{cap}$$

where *P*_*cap*_ denotes the capillary pressure. Young-Laplace equation is applied for calculating capillary pressure.

The following mass balance equations are also satisfied at equilibrium^37,49,50^,

6 $$F{z}_{i}={x}_{i}L+{y}_{i}V,\,i=1,\ldots ,{N}_{c},$$

7 $${\sum }_{i=1}^{Nc}{x}_{i}={\sum }_{i=1}^{Nc}\,{y}_{i}=1,$$

8 $$\sum _{i=1}^{Nc}\frac{({K}_{c}^{i}-1){z}_{i}}{1+\alpha ({K}_{c}^{i}-1)}=0,$$

where *z*_*i*_ is the overall mole fraction of component *i*. *x*_*i*_ and *y*_*i*_ are liquid and vapor compositions, respectively. *N*_*c*_ is the number of components in the system. *F* is the number of moles of original feed. *L* and *V* are the number of moles of liquid and vapor phases, respectively. ${K}_{c}^{i}$ is the equilibrium constant considering the capillary pressure. Wilson’s equation is applied to estimate the initial equilibrium constant^51^. The equilibrium constant is updated based on ratio of fugacity coefficients, which is evaluated as ${\varphi }_{L}^{i}/{\varphi }_{V}^{i}$. ${\varphi }_{L/V}^{ii}$ is the fugacity coefficient of component *i* in the liquid or vapor phase.

The fugacity coefficients can be obtained from Peng-Robinson equation of state^52^

9 $$P=\frac{RT}{{V}_{m}-b}-\frac{a(T)}{{V}_{m}^{2}+2b{V}_{m}-{b}^{2}},$$

where *V*_*m*_ is the mole volume of component *i*, *R* is the universal gas constant. *a*(*T*) and *b* are the parameters calculated using van der Waals mixing rules.

Accounting for the difference between liquid and vapor pressure in Eqs (3) and (9) should be separately solved for liquid and vapor phases.

10 $${({Z}_{L})}^{3}-(1-{B}_{L}){({Z}_{L})}^{2}+({A}_{L}-2{B}_{L}-3{({B}_{L})}^{2}){Z}_{L}-({A}_{L}{B}_{L}-{({B}_{L})}^{2}-{({B}_{L})}^{3})=0,$$

11 $${({Z}_{V})}^{3}-(1-{B}_{V}){({Z}_{V})}^{2}+({A}_{V}-2{B}_{V}-3{({B}_{V})}^{2}){Z}_{V}-({A}_{V}{B}_{V}-{({B}_{V})}^{2}-{({B}_{V})}^{3})=0,$$

where ${A}_{L}=\frac{{a}_{L}\alpha {P}_{L}}{{R}^{2}{T}^{2}}$, ${B}_{L}=\frac{{b}_{L}{P}_{L}}{RT}$, ${A}_{V}=\frac{{a}_{V}\alpha {P}_{V}}{{R}^{2}{T}^{2}}$, ${B}_{V}=\frac{{b}_{V}{P}_{V}}{RT}$. *Z*_*L*_ and *Z*_*V*_ represent the compressibility of liquid and vapor phase, respectively. For root selection, the minimum root of Eq. (10) is determined for the liquid phase, while the maximum one of Eq. (11) is for the vapor phase. The roots satisfy the Gibbs free energy minimization.

Fugacity coefficients of different component in the liquid and vapor phases were also solved for different pressures, which are expressed as:

12 $$\begin{array}{rcl}\mathrm{ln}\,{\varphi }_{L}^{i} & = & \frac{{b}_{iL}}{{b}_{L}}({Z}_{L}-1)-\,\mathrm{ln}\,({Z}_{L}-{B}_{L})\\  &  & -\frac{{A}_{L}}{2\sqrt{2}{B}_{L}}(\frac{2\sum _{i=1}^{Nc}{x}_{jL}(1-{k}_{ij})\sqrt{{a}_{iL}{a}_{jL}}}{{a}_{L}}-\frac{{b}_{iL}}{{b}_{L}})\mathrm{ln}\,(\frac{{Z}_{L}+(\sqrt{2}+1){B}_{L}}{{Z}_{L}-(\sqrt{2}-1){B}_{L}}).\end{array}$$

13 $$\begin{array}{rcl}\mathrm{ln}\,{\varphi }_{V}^{i} & = & \frac{{b}_{iV}}{{b}_{V}}({Z}_{V}-1)-\,\mathrm{ln}\,({Z}_{V}-{B}_{V})\\  &  & -\frac{{A}_{V}}{2\sqrt{2}{B}_{V}}(\frac{2\,\sum _{i=1}^{Nc}{x}_{jV}(1-{k}_{ij})\sqrt{{a}_{iV}{a}_{jV}}}{{a}_{V}}-\frac{{b}_{iV}}{{b}_{V}})\mathrm{ln}\,(\frac{{Z}_{V}+(\sqrt{2}+1){B}_{V}}{{Z}_{V}-(\sqrt{2}-1){B}_{V}}).\end{array}$$

The Gibbs free energy criterion is applied for root selection. Newton-Raphson method is used to solve the set of nonlinear equations^53^. Fortran Language is used to accomplish this program. In this study, we only considered the effect of capillary pressure; other surface effects are neglected, which will be included in our future work.

**Figure 1:**
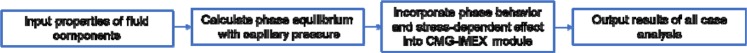
The flow chart of well performances in tight oil reservoirs.

In the following sections, we first use the aforementioned vapor-liquid phase equilibrium model to calculate the black-oil properties. The phase envelopes of different compositions were compared with the nanopore effects. Then, we built a field-scale reservoir simulation model using the CMG-IMEX simulator module^47^. Different PVT properties under the pore size distribution were included in the model. Finally, the impacts of nanopore effects and stress-dependent deformation were analyzed in detail. Figure 1 displays the flow chart of well performances in tight oil reservoirs. The main steps are summarized as follows:

Step 1: Input all properties of fluid component.

Step 2: Calculate the phase behavior considering capillary pressure. In this part, K-value initial estimates and capillary pressure are first evaluated; then solve Eqs (6) through (8). Afterwards, calculate compressibility factor and fugacity coefficients using Eqs (10) through (13). Check the fugacity coefficients with the convergence criteria.

Step 3: Incorporate the phase behavior results into CMG-IMEX module as well as stress-dependent deformation.

Step 4: Run simulation with CMG-IMEX simulator and obtain the results.

## Results and Discussion

### Phase behavior considering the effect of confined space

With the phase equilibrium model in “**Methodology**” Section, we investigated the effect of confined space on the bubble-point pressure and properties of the Bakken fluid. The P-T envelope of different fluid compositions was also calculated in this section. Table 1 lists the detailed compositions and properties of the Bakken fluid.

**Table 1:** Compositions and basic properties of the fluid.

Component	*z*_*i*_	*T*_*c*_, K	*P*_*c*_, atm	*V*_*c*_, L/mol	*w*_i_
C_1_	0.2506	190.60	45.40	0.0990	0.0080
C_2_–C_4_	0.2200	363.30	42.54	0.1970	0.1432
C_5_–C_7_	0.2000	511.56	33.76	0.3338	0.2474
C_8_–C_9_	0.1300	579.34	30.91	0.4062	0.2861
C_10+_	0.1994	788.74	21.58	0.9208	0.6869

**Figure 2:**
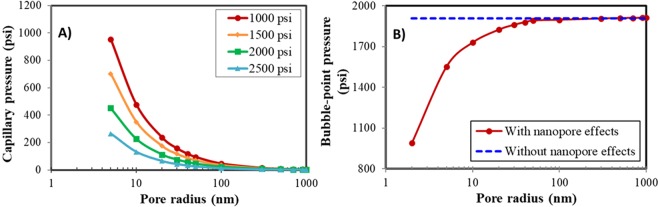
Capillary pressure and bubble-point pressure under different pore sizes. (**A**) Capillary pressure. (**B**) Bubble-point pressure.

We first calculated the capillary pressure and the bubble-point pressure for the Bakken fluid at different pore sizes. Figure 2 displays the capillary pressure and bubble-point pressure under different pore sizes. From Fig. 2(A), the smaller the pore radius, the larger the capillary pressure. When the pressure varies from 1000 psi to 2500 psi, the capillary pressure decreases, leading to less oil trapped in the nanopores. This is beneficial for the improvement of oil production. From Fig. 2(B), the nanopore effect is noticeable for the small pores (less than 50 nm). The reduction of pore sizes leads to the suppression of the bubble-point pressure. The nanopore effect can be neglected for the pores larger than 50 nm.

**Figure 3:**
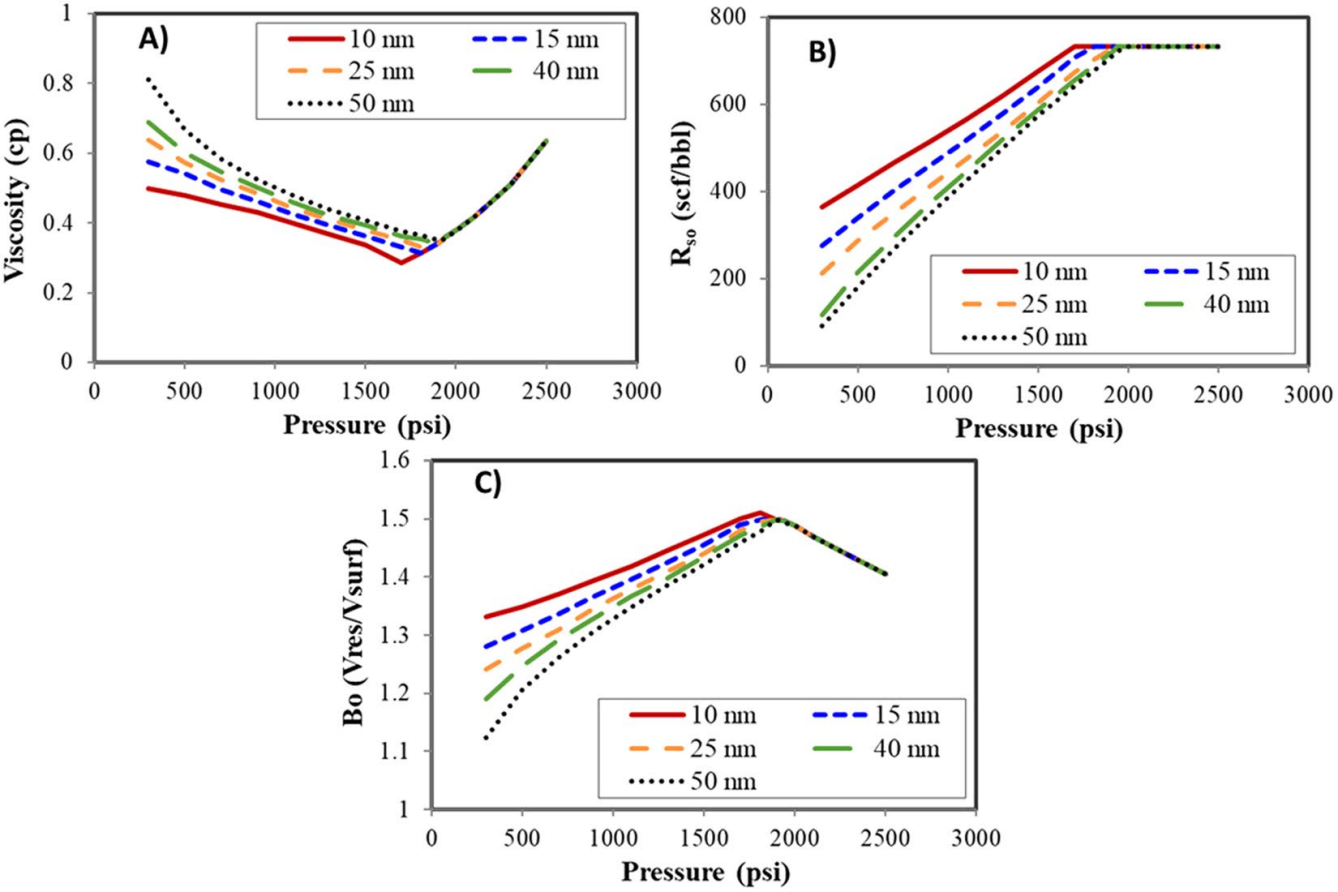
Black-oil properties under different pore sizes. (**A**) Oil viscosity. (**B**) Gas-oil ratio. (**C**) Formation volume factor.

Figure 3 illustrates the nanopore effect on the fluid black-oil properties. The viscosity, gas-oil ratio, and formation volume factor (FVF) were evaluated at the pore size of 10 nm, 15 nm, 25 nm, 40 nm, and 50 nm, respectively. The viscosity is calculated with the work by Fong and Nghiem^54^. The temperature is set as 240 °F.

As presented in Fig. 3, when the pore size reduces, gas-oil ratio and formation volume factor increases, while the oil viscosity decreases. The deviation is more significant at smaller pore sizes. The bubble-point pressure (inflection point) suppresses about 210 psi at the pore size of 10 nm compored to the bulk bubble-point pressure. Suppression of bubble-point pressure may lead to gas diffused in the oil phase at lower pressure. Once the pressure increases beyond the bulk bubble-point pressure, only single phase exists and the confined oil properties are the same with the fluid in the bulk phase. The properties at 50 nm are consistent with those without the nanopore effect, which is agree with the results in Fig. 2(B).

The impacts of fluid compositions were also included in this section. The fluid in Table 1 was applied, and we increased the percentage of each fluid’s original composition by 50%, one at a time, while adjusting the others accordingly, so that the total percentages add up to 100%. Table 2 lists the original and altered compositions of the Bakken fluid.

**Table 2:** The original and altered compositions of the Bakken fluid.

Compositions	Original	+50% C_1_	+50% C_2_–C_4_	+50% C_5_–C_7_	+50% C_8_–C_9_	+50% C_10+_
C_1_	0.2506	0.3759	0.2231	0.2256	0.2344	0.2257
C_2_–C_4_	0.2200	0.1887	0.3300	0.1950	0.2038	0.1951
C_5_–C_7_	0.2000	0.1687	0.1725	0.3000	0.1837	0.1750
C_8_–C_9_	0.1300	0.0986	0.1025	0.1050	0.1950	0.1051
C_10+_	0.1994	0.1681	0.1719	0.1744	0.1831	0.2991

**Figure 4:**
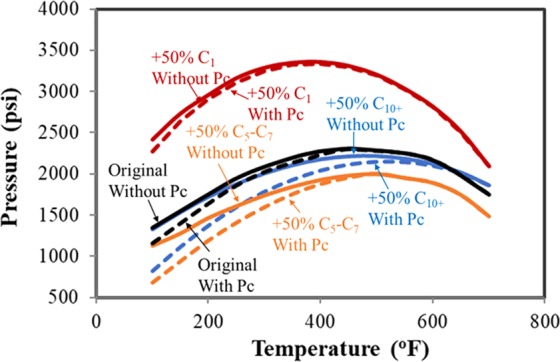
Pressure-temperature phase envelopes of different fluid compositions.

The effects of confinement were studied for the alteration of C_1_, C_5_–C_7_ and C_10+_, which represents light, mid-heavy and heavy components, respectively, as presented in Fig. 4. The P-T envelopes were compared with that of the original compositions.

From Fig. 4, it can be seen that bubble-point pressure decreases and the envelope considering the confinement deviates from the case without the confinement effect. At the temperature of 240 °F, the suppression of bubble-point pressure is 70 psi, 210 psi, and 300 psi for the increase of C_1_, C_5_–C_7_, and C_10+_, while the suppression is 130 psi for the original compositions. The increase in C_10+_ results in the largest shrinkage of two-phase region and the suppression of the bubble-point pressure, while the effect of C_1_ component is weak on the P-T phase envelope. When the reservoir condition approaches to the critical point, the impacts can be neglected. Hence, change of composition causes noticeable difference in the phase behavior, especially the condition away from the critical point. These observations agree well with the work taken by Nojabaei *et al*.^14^.

### Field-scale reservoir simulation model

West *et al*. (2013) have pointed out that lateral length in the Bakken in most cases has two scenarios, one is “short laterals” with approximately one mile long and spaced at 640 acres; the other is “long laterals” with approximately two miles long with spaced at 1280 acres^55^. In addition, Yu and Sepehrnoori have optimized the well spacing and pointed out that 4 horizontal wells are often drilled in a unit of 1280 acres (1 mile × 2 miles)^56^. Hence, in this section, we investigated a quarter of the field model with the dimensions of 5280 ft × 2640 ft × 50 ft, which corresponds to length, width, and height, respectively. The grid block is decided as 40 ft × 40 ft × 50 ft in *x*, *y*, and *z* directions, respectively, which can maintain balance of accuracy and computation speed. It covers an area of approximately a quarter section. Two horizontal wells with the length of 4,640 ft are set in the model. The well spacing is 1,320 ft. There are 15 hydraulic fractures along each well. Fracture spacing is 320 ft and fracture height is 50 ft. The reservoir temperature and initial pressure remain 240 °F and 7800 psi, respectively. The bottomhole pressure (BHP) of 1500 psi is set as the simulation constraint. The detailed properties for the reservoir and fracture properties are shown in Table 3, which are in the reasonable range of characteristics of Bakken tight formation^57^.

**Table 3:** Reservoir and fracture properties from middle Bakken for simulation study.

Properties	Value	Unit
Reservior temperature	240	°F
Initial reservoir pressure	7500	psi
Water saturation	49%	—
Total compressibility	1 × 10^−6^	psi^−1^
Porosity	5.6%	—
Number of fractures	30	—
Fracure width	0.01	ft
Fracture spacing	320	ft

The fluid compositions and properties of five pseudo components have been shown in Table 1. GOR is 1000 SCF/bbl, oil gravity is 42°API, and oil formation factor is 1.6. The properties above are within the reasonable range of typical values for the Bakken formation.

The history matching was conducted to calibrate the fracture parameters for the simulation study. During the history matching, oil flow rate was used as the simulation constraint, while the bottomhole pressure and gas flow rate were the history-matching variables. Matrix permeability, fracture half-length, and fracture conductivity were the main tuning parameters.

**Figure 5:**
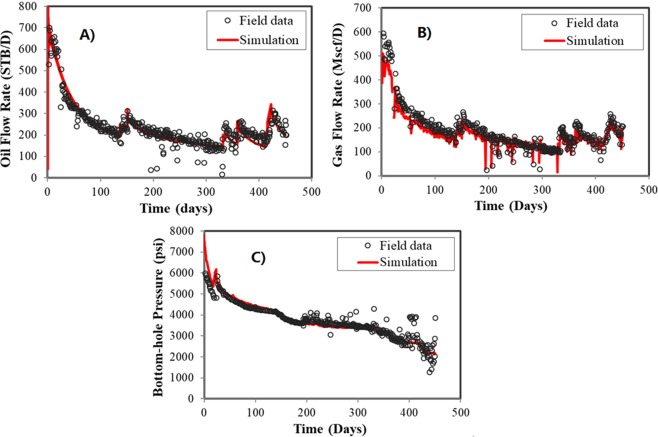
History matching results for the field case study with confinement effect. (**A**) Oil flow rate. (**B**) Gas flow rate. (**C**) Bottomhole pressure.

**Figure 6:**
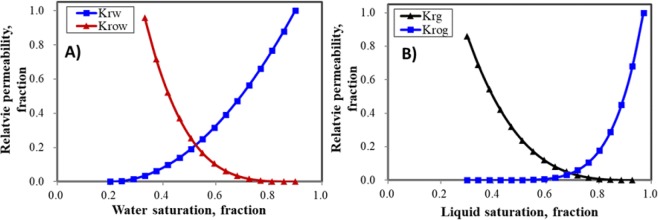
Relative permeability curves. (**A**) Water-oil relative permeability curve. (**B**) Liquid-gas relative permeability curve.

Figure 5 dispalys the history matching results for the field case study with confinement effects. The field data is obtained from an actual well of the Bakken tight oil reservoirs. By the history matching, fracture half-length of 300 ft and fracture conductivity of 50 mD·ft are determined considering the effects of confined space. Relative permeability curves are presented in Fig. 6.

**Figure 7:**
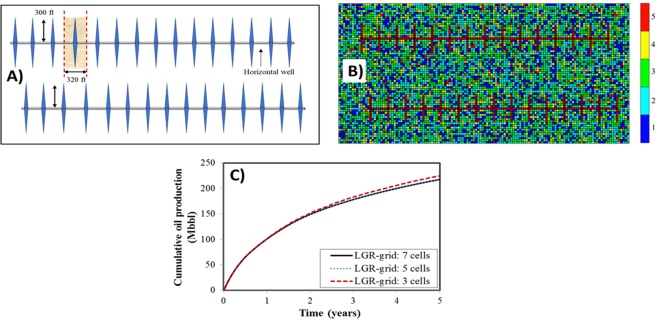
Schematic reservoir simulation model with the pore size distribution and the effect of LGR-grid sizes. (**A**) Schematic reservoir simulation model. (**B**) Pore size distribution (Color bars of 1–5 represent five different regions). (**C**) Effect of LGR-grid sizes on the well performance.

The schematic reservoir simulation model and the pore size distribution are shown in Fig. 7. According to the nanopore size distribution of a rock sample from Middle Bakken formation and the frequency of each pore size^6^, we divided the pore sizes into different regions. It should be noted that the division of pore size distribution is based on the experimental measurements of the tight rock. We performed the sensitivity analysis to identify the computational time and accuracy of different patterns. Finally, we found that distribution into five regions is a better choice to maintain balance of accuracy and computation speed. Five regions include: smaller than 10 nm (27%), 10–20 nm (26%), 20–30 nm (30%), 30–50 nm (13%), and larger than 50 nm (4%), as shown in Fig. 7(B). Since different pore sizes in this model have their own PVT properties and permeabilities, we applied the black-oil properties under 10 nm, 15 nm, 25 nm, 40 nm, and 50 nm (Fig. 3) to represent different regions.

During the simulation, the local grid refinement (LGR) method was utilized to model hydraulic fracture explicitly, which can accurately describe the transport from matrix to fracture. In order to avoid the dispersion effect induced by refined cells in modeling hydraulic fractures, we compared the cumulative oil production in 5 years with the number of refined cells ranging from 3 to 7. Figure 7(C) displays the effects of LGR-grid size on the well performances. From Fig. 7(C), the results of LGR with 5 cells are similar with 7 cells. Therefore, 5 cells are appropriate to diminish the numerical dispersion effect, which is applied in the following case studies.

### Field-scale case studies

In this section, we evaluated the impacts of confined phase behavior and stress-dependent deformation on the well performances. The simulation model in Section 3.2 is set as the base case for the following case studies. Reservoir size and fracture properties are kept the same, as shown in Fig. 7. The permeability and fracture conductivity under different pore sizes were calculated, which were implemented into the black-oil reservoir simulator CMG-IMEX module.

During the depletion process, stress changes and influences the matrix permeability and fracture conductivity of the tight oil reservoirs. Civan demenstrated that Carman-Kozeny equation of permeability was appliable in shale^58^; hence, the corresponding permeability can be calculated based on Carman-Kozeny equation and Nelson’s correlation^59–61^.

Table 4 lists the permeability and capillary pressure under different pore sizes, which are expected to be close to the practical situation.

**Table 4:** Permeability and capillary pressure under different pore sizes.

Pore radius (nm)	50	40	30	20	10
Permeability (md)	0.0079	0.0058	0.0036	0.0024	0.0010
P_cap_ (psi)	101.52	123.28	159.54	246.56	493.11

Geomechanics is complex in the tight formations. It negatively impacts the fluid flow capacity in the porous media. Alramahi and Sundberg fitted the laboratory measurement data and presented the relationship between normalized fracture conductivity and closure stress as below^62^.

14 $${\rm{Stiff}}\,{\rm{Shale}}:\,\mathrm{log}\,({F}_{c})=-\,0.0001\times \sigma -0.1082,\,{R}^{2}=0.954$$

15 $${\rm{Medium}}\,{\rm{Shale}}:\,\mathrm{log}\,({F}_{c})=-\,0.0004\times \sigma +0.2191,\,{R}^{2}=0.998$$

16 $${\rm{Soft}}\,{\rm{Shale}}:\,\mathrm{log}\,({F}_{c})=-\,0.0006\times \sigma -{\rm{0.4256}},\,{R}^{2}=0.987$$

which *F*_*c*_ is fracture conductivity, *σ* is the closure stress, defining as the difference between the minimum horizontal stress and bottom hole pressure. In this case, stiff shale is selected to investigate the impact of geomechanics in the Middle Bakken formation. The stress-dependent fracture conductivity is generated using Eq. (10), as shown inFig. 8.

**Figure 8:**
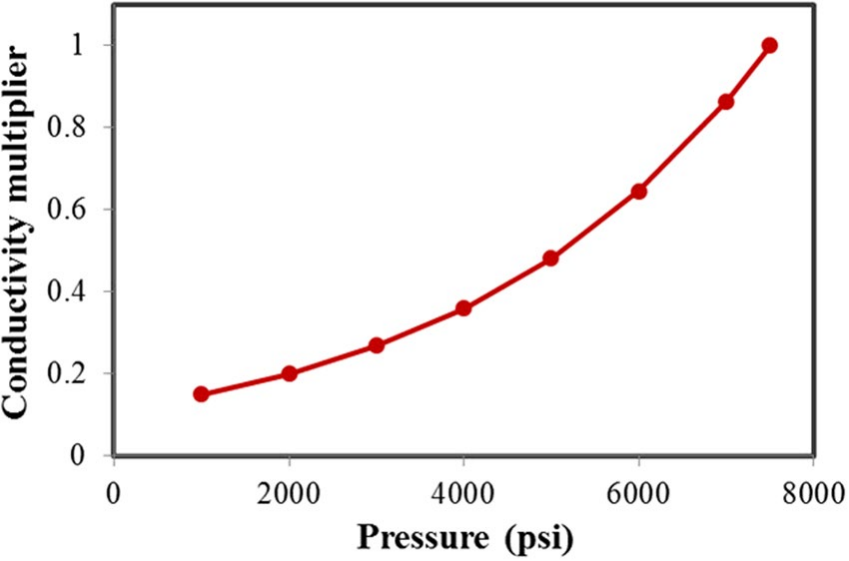
Stress-dependent conductivity multiplier of rock sample from the Bakken formation.

The well production was forecasted to investigate the effects of confinement and stress-dependent deformation during 30 years. Three cases are shown as follows,

(A) **Case A**: Without effect of confinement and stress-dependent deformation;
(B) **Case B**: With effect of confinement but without stress-dependent deformation;
(C) **Case C**: With effect of confinement and stress-dependent deformation.

**Figure 9:**
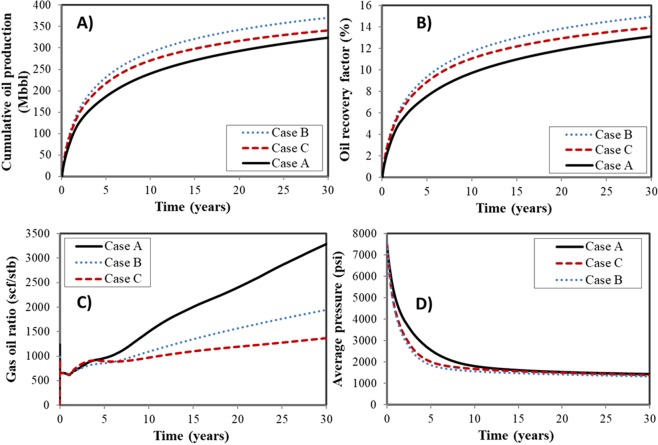
Well production of three cases in a 30-year period. (**A**) Cumulative oil production. (**B**) Oil recovery factor. (**C**) Gas oil ratio. (**D**) Average pressure.

Figure 9 presents the well performance during 30-year periods. As shown in Fig. 9(A,B), Case B produces the most oil from the Bakken tight oil reservoir, increasing by 14.3% of the cumulative oil production and 2.1% of the oil recovery factor. The effect of confinement on phase equilibrium favors the oil transport from matrix to factures, then to wellbore. Additionally, the confinement induces the reduction of the oil viscosity, leading to the increase of oil mobility and oil production.

The results in Case C indicate that the deformation decreases rock permeability and negatively influences the fluid flow capacity. The stress-dependent deformation affects the connections, leading to the resistance during the fluid flow process. However, it should be noted that with the consideration of rock deformation, pore size reduces, resulting in more suppression of the bubble point pressure for the confined fluid during the oil production. The two-phase flow delays with light components dissolved in it, and the flow capacity increases. Hence, it would be more complex when considering the rock deformation. Two effects of increasing effect of confinement and decreasing permeability compete with each other.

Figure 9(C) displays the gas oil ratio (GOR) for different cases. Due to the early production mainly from fractures, GOR increases at very early period and then quickly decreases before steady increase. As shown, the GOR for the case considering confinement effect (Case B) is lower than the case without confinement effect (Case A). The GOR for the case with confinement effect and stress-dependent deformation (Case C) exhibits an increase at early time and reduce later. The reason might be that the stress-dependent deformation caused the reduction in the pore sizes and the further suppression of bubble-point pressure. Hence, more hydrocarbons will flow from matrix to the wellbore in the liquid phase. The difference of GOR also impacts the oil production.

Figure 9(D) illustrates the average pressure during the depletion process. It can be clearly seen that the pressure of Case B experiences the largest decrease in the 30-year period. The large pressure drop indicates that the well performance will be overestimated if stress-dependent deformation is ignored.

**Figure 10:**
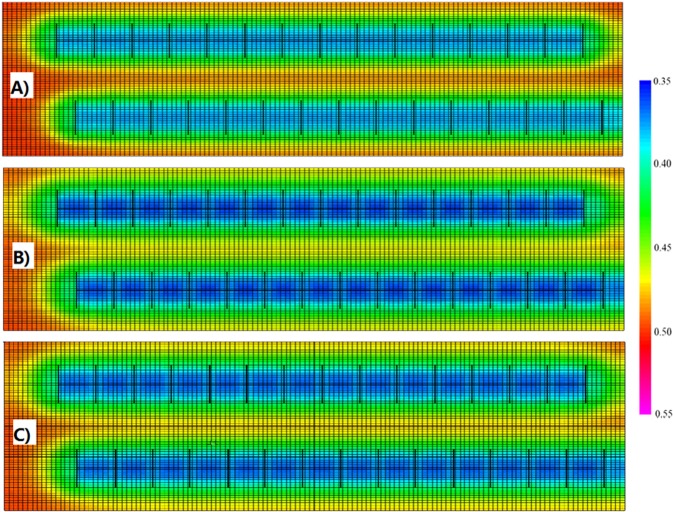
The oil saturation at the end of production for three cases. (**A**) Case A. (**B**) Case B. (**C**) Case C.

Figure 10 displays the oil distribution at the end of 30 years. The oil saturation in Case B is the lowest, while the oil saturation in Case A is the largest. During the process of production, the oil around the fractures has been produced gradually, and oil is trapped in the regions away from the fractures. Additionally, the oil between two horizontal wells is also swept in Case B and Case C, leading to higher oil production.

## Conclusions

1. An efficient numerical model of evaluating well performance considering the confinement effects on the phase behavior and stress-dependent deformation was developed.
2. For the fluid compositions like Bakken oil, the phase behavior in confined space significantly deviates from the bulk fluid. The change of heavy components brings large deviation on the phase envelope.
3. Simulation studies of confinement effect and stress-dependent deformation on the well production of Bakken tight oil reservoirs were conducted. The results indicate that the effect of confinement on phase equilibrium leads to 14.3% of cumulative oil production and 2.1% of oil recovery factor, respectively, for these typical reservoirs.
4. The stress-dependent deformation decreases the rock permeability and impacts the flow capacity, leading to the suppression of the cumulative oil production and oil recovery factor. Simulation results of oil production in tight oil reservoirs are demonstrated to sensitive to rock compaction during reservoir depletion. Therefore, it should be included in the simulation analysis.
5. This simulation study provides a useful approach, incorporating phase behavior calculation and the reservoir simulator to investigate the factors affecting the well production. This comprehensive model can be easily used to evaluate the effects of confinement and stress sensitivity on the well performance of tight oil reservoirs.
